# Effect of Topical Silver Nanoparticle Formulation on Wound Bacteria Clearance and Healing in Patients With Infected Wounds Compared to Standard Topical Antibiotic Application: A Randomized Open-Label Parallel Clinical Trial

**DOI:** 10.7759/cureus.60569

**Published:** 2024-05-18

**Authors:** Basanti K Pathi, Subrajit Mishra, Niranjan Moharana, Abinash Kanungo, Amaresh Mishra, Subrat Sahu, Rajesh K Dash, Rajan Dubey, Manoja K Das

**Affiliations:** 1 Department of Microbiology, Kalinga Institute of Medical Sciences, Bhubaneswar, IND; 2 Department of Surgery, Kalinga Institute of Medical Sciences, Bhubaneswar, IND; 3 Department of Public Health and Technology, Health Technology Consultant, Bhopal, IND; 4 Department of Public Health, The INCLEN Trust International, New Delhi, IND

**Keywords:** ­wound healing, bacteria clearence, antibiotic sparing, wound care treatment, silver nanoparticle

## Abstract

Background: Infected wounds pose a special challenge for management, with an increased risk of wound chronicity, systemic infection, and the emergence of antibiotic resistance. Silver nanoparticles have multimodal effects on bacteria clearance and wound healing. This study aimed to document the efficacy of a topical silver nanoparticle-based cream on bacteria clearance and wound healing in infected wounds compared to Mupirocin.

Methods: This open-label parallel randomized clinical trial allocated 86 participants with infected wounds (culture-positive) into Kadermin, silver nanoparticle-based cream arm (n=43) and Mupirocin arm (n=43) and documented the swab culture on day 5 and wound healing at day 28, along with periodic wound status using the Bates-Jensen Wound Assessment Tool. Patients received oral/systemic antibiotics and other medications for underlying diseases. The intention-to-treat principle was adopted for data analysis using the chi-square and Student t tests to document the differences between groups according to variable characteristics.

Results: All participants completed the follow-up. On day 5, wound bacteria clearance was observed in 86% and 65.1% of the participants in the Kadermin and Mupirocin arms, respectively (p=0.023). At day 28, complete wound healing was observed in 81.4% and 37.2% of the participants in the Kadermin and Mupirocin arms, respectively (p≤0.001). No local or systemic adverse event or local reaction was observed in any of the participants.

Conclusion: Kadermin, the silver nanoparticle-based cream, has better efficacy in achieving faster wound bacteria clearance and healing in infected wounds compared to Mupirocin. This may have relevance for its use as an antibiotic-sparing agent in wound management.

## Introduction

Antimicrobial resistance-attributable deaths are expected to rise from 700,000 in 2014 to 10 million by 2050, with US$100 trillion in lost output [1]. India carries the largest burden of drug-resistant organisms globally [2]. With multidrug-resistant (MDR) and extensively drug-resistant (XDR) bacterial infections, the mortality risk increases multifold depending on the degree of resistance, the organism, and associated risk factors [3]. Acute and chronic wound infections are significant problems, and 20% of surgical sites and 40% of diabetic wounds infected with resistant bacteria have been reported from India [4,5]. The common organisms isolated from the wound infections are *Staphylococcus aureus*, *Escherichia coli*, *Pseudomonas aeruginosa*, *Acinetobacter baumannii*, and *Klebsiella pneumonia* [5].

Silver is a broad-spectrum antimicrobial agent with activity against bacteria, fungi, and yeast that has been in different forms and formulations for a long time [6]. Several metal nanoparticles have demonstrated effects on Gram-positive and Gram-negative bacteria in experimental models, animals, in vitro, and preliminary human studies [7]. Given the benefits, many silver (Ag)-containing wound dressings have been authorized by the US Food and Drug Administration.

The global wound care market is projected to grow at a compound annual growth rate (CAGR) of 6.3% between 2023 and 2030 (from USD 19.63 billion in 2023 to USD 30.04 billion by 2030) [8]. The silver nanoparticles market is projected to grow at a CAGR of 15.6% during 2021-2030 (from USD 1.5 billion in 2020 to USD 6.6 billion by 2030) [9]. The global antimicrobial additives market is projected to grow at a CAGR of 7.1% during 2021-2030 (from USD 4.7 billion in 2020 to USD 9.3 billion by 2030) [10].

The nanoparticles, with their bactericidal properties, provide an opportunity for use as antibiotic-sparing agents in treating resistant wound infections and minimizing antibiotic resistance [11-13]. While the market for silver nanoparticle-based products is rising, the evidence from India is limited, which mandates documentation of its effect in different types of wounds and settings. If found successful, nanoparticle production and usage may revolutionize infected wound management, which will be very useful in resource-poor settings.

This study compared the efficacy of the topical application of Kadermin, a silver nanoparticle-containing cream, on wound microbial clearance and healing to that of an antibiotic cream in patients with infected wounds. In this study, we used Mupirocin ointment for comparison with the commonly used topical antibiotics in the surgical ward where the study was conducted.

## Materials and methods

Design

This was a prospective, open-label, randomized, parallel-design clinical trial.

Participants and settings

This study was conducted at one tertiary care hospital in Odisha, India, after obtaining institutional ethical committee approval. Patients of any gender, aged above 18 years, with infected wounds (any bacteria isolated from the swab or pus discharge by standard culture) of any type, location, duration, and size, irrespective of underlying co-morbid condition, and both hospitalized and ambulatory patients, were included. Considering the feasibility, the patients agreeing to attend the hospital for assessment and dressing as per the proposed follow-up schedule were included. We excluded pregnant or breastfeeding women, patients with underlying cancer-related wounds, those with immunocompromised status, those requiring life support or vasopressors, and those involved in any other clinical study.

Recruitment and randomization

We approached the patients who met the eligibility criteria for consent, using the patient information and consent form in the local language. After obtaining consent, the participants were recruited and randomized into one of the two arms in a 1:1 ratio using serially numbered, triple-layered opaque envelopes bearing unique IDs. The randomization sequence was generated using variable blocks 4 and 6. The sealed envelopes were prepared and supplied by an independent biostatistician and kept by an independent nurse. After obtaining consent and recruitment, the study coordinator obtained the envelope bearing the same serial number as the recruited participant and opened it for arm allocation.

Investigational products

The participants allocated into the intervention arm (arm 1) were given Kadermin cream (containing SCX powder 1.5% consisting of silicon dioxide 75-85%, chlorhexidine 15.4-25.14%, silver 0.14-0.40%, hyaluronic acid, sodium salt 0.20%, dimethicone 0.5%, and emulsifying agents) for topical application on the wounds (Supplement Table 4). The participants allocated into the comparison arm (arm 2) were given Mupirocin ointment (2% containing mupirocin 20 mg USP; bland water miscible ointment base with polyethylene glycol 400 and polyethylene glycol 3350) for topical application on the wounds. These products were provided free of charge to the participants.

Wound and clinical care

The participants were given standard wound care and dressing by the surgeon, including cleaning, debridement, and exudate cleaning, followed by topical application of Kadermin or Mupirocin cream/ointment on the wound at a 1 g/cm^2^ dosage daily until wound healing or 28 days, whichever was earlier. The topical application and wound dressing continued until wound healing was assessed by the treating surgeon. The investigational products were used until wound healing was achieved or the patient was taken up for advanced wound care. The patients received systemic (oral and/or injectable) medications, including antibiotics and other medications, if needed, as per the treating doctor’s assessment. The antibiotic sensitivity test (AST) was done for all the isolates from wounds using VITEK-2 cards according to the bacteria types isolated. The systemic antibiotics were initiated as per the hospital antibiotic policy and modified subsequently as per the AST results by the treating surgeons.

Measurements and documentation

During the dressing, wound status and characteristics were assessed using the Bates-Jensen Wound Assessment tool, and scores were recorded [14] (Supplement Table 5). The wound photographs were taken before and after dressing, and wound size (mean surface area, MSA) was measured on days 0, 3, 5, 10, 14, and 28. A repeat swab for bacterial culture was sent on day 5 (+1 day). Fever and local pain (using a Visual Analogue score of 0-10), tenderness, and redness were documented. The antibiotics and other drugs given and dressing materials used were documented. Any adverse event (local or systemic) was documented. The surgeon performed the wound dressing and measurements in accordance with the follow-up schedule. The ambulatory patients visited the hospital as per the schedule for wound assessment and dressing by the surgeon. Depending on the type of wound, the surgeon made more frequent dressings for some patients.

Study outcomes

The primary outcome was the proportion of patients achieving wound microbiological clearance on day 5 (in the swab/discharge from the wound bed). The secondary outcomes were the proportion of patients with wound healing and wound size on days 5 and 10, the median time (in days) needed for wound healing, and any features of major toxicity or adverse events that warranted the stoppage of the therapy. Wound healing was documented by clinician examination for re-epithelialization.

Data analysis

Analyses of this study were carried out based on the intention-to-treat principle. The chi-square test and Student's t-test were used to determine the differences in baseline characteristics between the groups for categorical variables and continuous variables. To examine the potential influence of potential effect modifiers like co-morbidity, wound size, bacteria isolated, and gender were considered for stratified analysis and logistic regression analysis. The significance level was set at 0.05.

Sample size

Assuming an anticipated day 5 microbial clearance in 50% of the patients in the Kadermin arm compared to 18% in the Mupirocin arm, the desired sample size at a 95% confidence level with 80% power was estimated to be 40 per arm. We planned to recruit 50 patients per arm, a total of 100 patients.

Ethical issues

The study protocol was reviewed and approved by the institutional ethical committee and conducted in conformity with ICH-GCP guidelines, the Helsinki Declaration, and the local regulatory requirements (Indian GCP, Indian Council of Medical Research, and New Drugs and Clinical Trials Rules, 2019). The protocol was registered on the Clinical Trials Registry-India (www.ctri.nic.in) before the enrolment of the first participant (CTRI/2022/03/041120).

## Results

**Figure 1:**
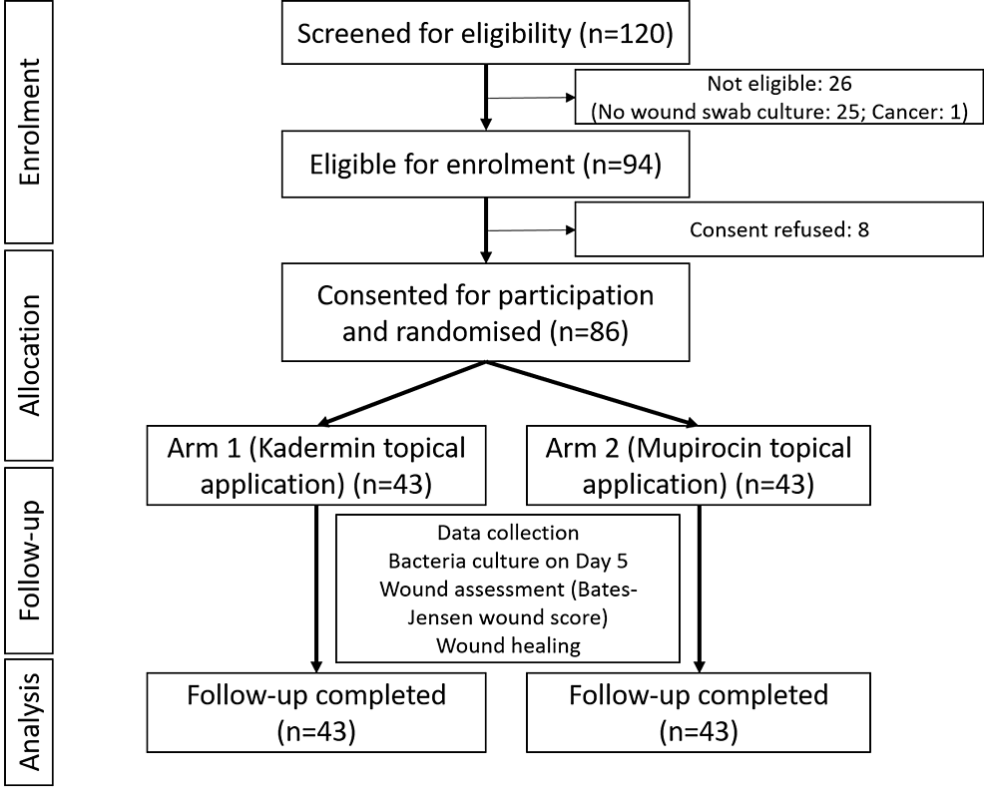
Participant recruitment and follow up flow chart

During the study period (March-December 2022), 86 patients were recruited, and 43 each were randomized into two arms. Figure 1 depicts the participant flow chart for enrollment and follow-up. There were no wound care-related complications, and all participants received their assigned medication. No crossover between the arms or loss of follow-up was observed.

The baseline demographic data and wound characteristics were similar between the arms (Table 1). The study sample was predominantly male in both arms with similar ages. Most of the ulcers were located in the lower limbs, below the ankles. The associated clinical symptoms were comparable. Most of the patients had single bacteria isolated in both groups.

**Table 1:** The baseline characteristics of the participants Note: n: number of observations; SD: standard deviation

Parameter	Kadermin (n=43)	Mupirocin (n=43)	P-value
Demography			
Gender: Male, n (%)	36 (83.7)	32 (74.41)	0.289
Age in years, mean (SD)	51.46 (14.1)	56.93 (14.78)	0.082
Location of the wound			
Upper limb/hands, n (%)	4 (9.3)	3 (7.0)	0.716
Lower limbs/legs, n (%)	36 (83.7)	37 (86.0)	
Other sites, n (%)	3 (7.0)	3 (7.0)	
Wound size			
Surface area (in sq cms), mean (SD)	12.6 (11.2)	13.6 (11.8)	0.687
Associated symptoms			
Fever, n (%)	9 (20.9)	9 (21.0)	0.971
Pain, n (%)	39 (90.7)	39 (90.7)	0.368
Pan score, mean (SD)	4.4 (1.5)	4.4 (1.5)	1.000
Bleeding, n (%)	19 (44.2)	15 (34.9)	0.667
Pus discharge, n (%)	25 (58.1)	26 (60.5)	0.680
Swelling, n (%)	32 (74.4)	31 (72.0)	0.948
Bacteria isolation			
Single bacteria isolate, n (%)	40 (93.0)	39 (90.7)	0.693
Two bacteria isolate, n (%)	3 (7.0)	4 (9.3)	
Co-morbidity			
Diabetes mellitus	9 (20.9)	8 (18.6)	0.574
Treatment given			
Systemic/oral antibiotics given, n (%)	31 (72.0)	30 (69.8)	0.812

The bacteria isolated from the wounds of the participants at baseline are summarized in Table 2. *Staphylococcus aureus*, *Klebsiella pneumoniae*, *Escherechia coli*, and *Pseudomonas aeruginosa* were the commonly isolated bacteria from the wounds at baseline.

**Table 2:** The bacteria isolated at baseline from the wounds of the participants

Parameter	Kadermin, n (%)	Mupirocin, n (%)
Primary bacteria isolated		
*Acinetobactor baumani*	1 (2.3)	3 (7.0)
*Aeromonas hydrophila*	0 (0)	1 (2.3)
Citrobacter spp.	1 (2.3)	0 (0)
*Escherichia coli*	8 (18.6)	7 (16.3)
*Enterobacter cloacae*	1 (2.3)	1 (2.3)
*Enterococcus faecalis*	2 (4.7)	1 (2.3)
*Klebsiella pneumoniae*	9 (20.9)	9 (20.9)
*Proteus mirabilis*	4 (9.3)	3 (7.0)
*Pseudomonas aeruginosa*	6 (14.0)	7 (16.3)
*Pseudomobqs putida*	1 (2.3)	0 (0)
*Serratia marcescens*	0 (0)	1 (2.3)
*Staphylococcus aureus*	9 (20.9)	9 (20.9)
*Streptococcus pyogenes*	1 (2.3)	0 (0)
*Streptococcus sangoiris*	0 (0)	1 (2.3)
Second bacteria isolated		
*Enterobacter cloacae*	1 (2.3)	0
*Klebsiella pneumoniae*	0	2 (4.7)
*Proteus mirabilis*	1 (2.3)	1 (2.3)
*Pseudomobqs putida*	1 (2.3)	0
*Serratia marcescens*	0	1 (2.3)

The outcomes of day 5 and day 28 for the participants are presented in Table 3. After five days of therapy, 86% of the participants in the Kadermin arm achieved wound microbiological clearance compared to 65.1% in those in the Mupirocin arm, which was significant (p-value = 0.023). Compared to the Kadermin arm, more participants in the Mupirocin arm acquired different bacteria colonizations on subsequent cultures. Although there was a reduction in the wound size, pain score, and Bates-Jensen wound score in the Kadermin arm, no statistical significance was observed. On day 28, there was a significant difference in the wound healing status of the participants in the Kadermin arm (completely healed, 81.4%, and partially healed, 18.6%) compared to those in the Mupirocin arm (completely healed, 37.2%, and partially healed, 55.8%) (p-value = 0.000). We observed no local or systemic adverse events or local reactions in any of the arms. We observed no significant effect modification due to gender, comorbidity, wound size, or isolated bacteria.

**Table 3:** The outcome parameters for the participants at different time points Note: n: number of observations; SD: standard deviation

Parameter	Kadermin (n=43)	Mupirocin (n=43)	P-value
Wound bacteria colonization			
Day 0, n (%)	43 (100)	43 (100)	NA
Day 5, n (%)	6 (14.0)	15 (34.9)	0.023
Same bacteria isolated on day 5, n (%)	4 (9.3)	7 (16.3)	0.332
Different bacteria isolated on day 5, n (%)	2 (4.7)	8 (18.6)	0.435
Pain score			
Day 0, median (IQR)	5 (4, 6)	4 (3, 5)	0.927
Day 5, median (IQR)	3 (2, 4)	3 (2, 4)	0.170
Wound size (surface area in sq cms)			
Day 0, median (IQR)	8 (6, 12)	8 (6, 13)	0.687
Day 5, median (IQR)	6 (3, 8)	6 (4. 8)	0.916
Bates-Jensen wound score			
Day 0, median (IQR)	34 (32, 38)	35 (33. 37)	0.940
Day 5, median (IQR)	30 (26, 33)	30 (26, 34)	0.983
Outcome at day 28			
Would heal completely, n (%)	35 (81.4)	16 (37.2)	0.000
Would heal partially, n (%)	8 (18.6)	24 (55.8)	
No improvement, n (%)	0 (0)	3 (7.0)	

## Discussion

Management of chronic wounds and surgical site infections is a persisting challenge. Bacterial infection of wounds poses a significant challenge in management due to delays in wound healing, interventions and dressings needed, and antibiotic usage (topical and systemic, both duration and changes needed). Multiple antibiotic usage and a multi-bacteria wound milieu with topical antibiotic exposure further increase the risk of antibiotic resistance development. Thus, measures need to be identified that facilitate early bacterial clearance and encourage faster wound healing with antibiotic-sparing (systemic and/or topical).

This study documented higher wound bacteria clearance by day 5 with topical application of silver nanoparticle-based cream (Kadermin) compared to topical application of antibiotic ointment (Mupirocin) (86% vs. 65.1%, respectively, p = 0.023). Higher wound healing was also observed at day 28 with silver nanoparticle cream compared to the topical Mupirocin ointment (complete healing in 81.4% vs. 37.2%, respectively, p = 0.000). We could not retrieve any study that documented the impact on bacterial clearance of wounds with the use of the silver nanoparticle-based product in the literature.

The use of silver nanoparticles with thermoplastic polyurethane dressings among diabetic patients with open fractures significantly reduced bacteria colonization (4.88% vs. 12.28%), wound healing time, and hospitalization period [15].

A study using topical silver alginate powder significantly reduced the clinical infection score and had faster wound size/area after four weeks than foam dressing in adults with chronic wounds [16]. The use of silver nanoparticle dressing for pressure ulcers in spinal cord injury patients failed to demonstrate a significant difference in wound healing compared to hydrocolloid dressing, although the wound healing was better and faster [17]. 

The higher wound healing with silver nanoparticle-based application is consistent with the reported observations on the effectiveness of the use of various silver-based topical applications in different forms on wound healing, odor control, exudate reduction, and pain [18]. Although the wound healing process is affected by various factors, including the patient's nutritional status, age, co-morbidities, medication, occupation, and behavior, along with the size, depth, causation, and etiology of the wound, the effect of Kadermin was observed across different patients and various types of wounds. 

Biofilm formation is considered a critical factor in bacteria persistence and delay in wound healing, and bioactive silver products have been observed to reduce bacteria load [7,19,20].

Silver nanoparticles have antibacterial effects against both Gram-negative and Gram-positive bacteria, apart from fungi and viruses [21]. The silver nanoparticles have multimodal antimicrobial effects, including complex formation with DNA and RNA, enzyme inactivation, protein denaturation, ion exchange impairment, and cell membrane damage [22]. Apart from these antimicrobial effects, the silver nanoparticles also promote cell proliferation, growth factor release, and local immune and inflammatory modulation, apart from the antibacterial effects, which could explain the multimodal effects [23-26].

This study documented the effect on bacterial colonization of the wounds using the swab culture approach along with the wound healing outcome as hard outcomes instead of clinical parameters. However, this study has some limitations: cultures beyond day 5 were not collected, the mixed profiles of wounds of diverse etiology were included, and both hospitalized and ambulatory care patients were included. A smaller sample size could have limited documentation of the effect size for some parameters. The documentation of the effect on biofilm and tissue changes could not be done. The use of Mupirocin alone as the comparison product may be a limitation.

## Conclusions

Kadermin, the topical silver nanoparticle-based cream application, demonstrated better wound bacteria clearance and wound healing compared to the topical Mupirocin application in patients with infected wounds. The better antibacterial effect of silver nanoparticle-based products could offer an antibiotic-sparing option and contribute to antibiotic resistance containment. The mechanistic documentation of the effects of silver nanoparticles in different wound contexts may further improve understanding and clinical application.

## Appendices

**Table 4:** The composition and ingredients of Kadermin (sliver nanoparticles-based) cream

Name of the ingredients	Quantity (%)	Function
SCX-powder patented complex, including: silicon dioxide 75.0–85.0%; chlorhexidine 15.4–25.14%; silver 0.14–0.4%	1.50	Antimicrobial
Hyaluronic acid, sodium salt	0.2	Hydrating and film-forming agents
Dimethicone	0.5	Emollient, film-forming
D-panthenol	1	Moisturizer
Glycerin	3	Humectant
Steareth-21	2	Emulsifier
Steareth-2	3	Emulsifier
*Olea europaea* fruit oil	0.3	Emollient
Cetearyl ethylhexanoate	3	Emollient
Sweet almond oil	1.5	Emollient
Dicaprylyl ether	3	Emollient
AA mixture (Lys-Pro-Gly)	0.3 (0.1x3)	Skin restoring agent
*Aloe barbadensis* leaf extract	0.5	Moisturizer
SLM lamellar matrix (water/aqua, alcohol, caprylic/capric triglyceride, hydrogenated phosphatidylcholine, *Butyrospermum parkii* (shea) butter, squalane, ceramide NP)	0.5	Skin restoring agent
Xantan gum	0.3	Rheological modifier
Cetearyl alcohol	2	Emulsifier
*Butyrospermum parkii* butter	1.5	Emollient
Allantoin	0.3	Lenitive
Water	69.9	Solvent

**Table 5:** Bates-Jensen Wound Assessment Tool Reference [15]

Sl no.	Parameter		Observation/score
1	Grade according to size of the wound		
	1. Length × width: <4 sq cm	2. Length × width: 4 to <16 sq cm	
	3. Length × width: 16.1 to <36 sq cm	4. Length × width: 36.1 to <80 sq cm	
	5. Length × width: >80 sq cm	0. Not assessed	
2	Depth of the wound/ulcer		
	1. Non-blanchable erythema on intact skin		
	2. Partial thickness skin loss involving epidermis and/or dermis		
	3. Full thickness skin loss involving damage or necrosis of subcutaneous tissue; may extend down to but not through underlying fascia; and/or mixed partial and full thickness and/or tissue layers obscured by granulation tissue		
	4. Obscured by necrosis		
	5. Full thickness skin loss with extensive destruction, tissue necrosis or damage to muscle, bone or supporting structures		
	0. Not assessed		
3	Edges of the wound/ulcer		
	1. Indistinct, diffuse, none clearly visible	2. Distinct, outline clearly visible, attached, even with wound base	
	3. Well-defined, not attached to the wound base	4. Well-defined, not attached to the base, rolled under, thickened	
	5. Well-defined, fibrotic, scarred, or hyperkeratotic	0. Not assessed	
4	Undermining of the ulcer		
	1. None present	2. Undermining <2 cm in any area	
	3. Undermining 2-4 cm involving <50% wound margins	4. Undermining 2–4 cm involving >50% wound margins	
	5. Undermining >4 cm or tunneling in any area	0. Not assessed	
5	Presence of necrotic tissue and type		
	1. None visible	2. White/gray non-viable tissue and/or non-adherent yellow slough	
	3. Loosely adherent yellow slough	4. Adherent, soft, black eschar	
	5. Firmly adherent, hard, black eschar	0. Not assessed	
6	Necrotic tissue amount		
	1. None visible	2. <25% of wound covered	
	3. >25 to <50% of wound covered	4. >50% to <75% of wound covered	
	5. 75% to 100% of wound covered	0. Not assessed	
7	Type of exudate present		
	1. None	2. Bloody	
	3. Serosanguinous: thin, watery, pale red/pink	4. Serous: thin, watery, clear	
	5. Purulent: thin or thick, opaque, tan/yellow, with or without odor	0. Not assessed	
8	Amount of the exudate		
	1. None, dry wound	2. Scant, wound moist but no observable exudate	
	3. Small	4. Moderate	
	5. Large	0. Not assessed	
9	Skin color surrounding the wound		
	1. Pink or normal for ethnic group	2. Bright red and/or blanches to touch	
	3. White or grey pallor or hypopigmented	4. Dark red or purple and/or non-blanchable	
	5. Black or hyperpigmented	0. Not assessed	
10	Peripheral tissue edema		
	1. No swelling or edema	2. Non-pitting edema extends <4 cm around the wound	
	3. Non-pitting edema extends >4 cm around the wound	4. Pitting edema extends <4 cm around the wound	
	5. Crepitus and/or pitting edema extends >4 cm around the wound	0. Not assessed	
11	Peripheral tissue induration		
	1. None present	2. Induration, <2 cm around wound	
	3. Induration 2-4 cm extending <50% around the wound	4. Induration 2-4 cm extending >50% around the wound	
	5. Induration >4 cm around wound	0. Not assessed	
12	Granulation tissue status		
	1. Skin intact or partial thickness wound	2. Bright, beefy red; 75% to 100% of wound-filled and/or tissue overgrowth	
	3. Bright, beefy red; <75% and >25% of wound filled	4. Pink and/or dull, dusky red, and/or fills < 25% of wound	
	5. No granulation tissue present	0. Not assessed	
13	Epithelialization status		
	1. 100% wound covered, surface intact	2. 75% to <100% wound-covered and/or epithelial tissue extends >0.5 cm into the wound bed	
	3. 50% to <75% wound covered and/or epithelial tissue extends to <0.5 cm into the wound bed	4. 25% to <50% wound covered	
	5. <25% wound covered	0. Not assessed	
	Bates-Jensen Wound Score (total)		
